# Protein *O*- and *C*-Glycosylation pathways in *Toxoplasma gondii* and *Plasmodium falciparum*

**DOI:** 10.1017/S0031182019000040

**Published:** 2019-02-18

**Authors:** Giulia Bandini, Andreia Albuquerque-Wendt, Jan Hegermann, John Samuelson, Françoise H. Routier

**Affiliations:** 1 Department of Molecular and Cell Biology, Boston University, Goldman School of Dental Medicine, 72 East Concord Street, Boston, MA 02118, USA; 2 Department of Clinical Biochemistry OE4340, Hannover Medical School, Carl-Neuberg-Strasse 1, 30625 Hannover, Germany; 3 Hannover Medical School, Electron Microscopy Facility OE8840, Carl-Neuberg-Strasse 1, 30625 Hannover, Germany

**Keywords:** Mucin type *O*-glycosylation, nucleocytosolic *O*-glycosylation, thrombospondin type 1 repeat, *O*-fucosylation, *C*-mannosylation, *Toxoplasma gondii*, *Plasmodium falciparum*, Apicomplexa

## Abstract

Apicomplexan parasites are amongst the most prevalent and morbidity-causing pathogens worldwide. They are responsible for severe diseases in humans and livestock and are thus of great public health and economic importance. Until the sequencing of apicomplexan genomes at the beginning of this century, the occurrence of *N*- and *O*-glycoproteins in these parasites was much debated. The synthesis of rudimentary and divergent *N*-glycans due to lineage-specific gene loss is now well established and has been recently reviewed. Here, we will focus on recent studies that clarified classical *O*-glycosylation pathways and described new nucleocytosolic glycosylations in *Toxoplasma gondii,* the causative agents of toxoplasmosis. We will also review the glycosylation of proteins containing thrombospondin type 1 repeats by *O*-fucosylation and *C*-mannosylation, newly discovered in *Toxoplasma* and the malaria parasite *Plasmodium falciparum.* The functional significance of these post-translational modifications has only started to emerge, but the evidence points towards roles for these protein glycosylation pathways in tissue cyst wall rigidity and persistence in the host, oxygen sensing, and stability of proteins involved in host invasion.

## Introduction

Infections by unicellular protozoan parasites are a major worldwide health concern. It is estimated that parasitic diseases cause more than 1 million deaths every year and billions endure the morbidity of infection. Infections caused by intracellular parasites of the phylum Apicomplexa are the most prevalent, severely compromise human health, and impact animal food production. The most medically important Apicomplexa are the *Plasmodium* species, the causative agents of malaria, and in particular *Plasmodium falciparum,* which is responsible for most malaria-related deaths (WHO, 2018). Increased prevention and control measures have led to a marked reduction in malaria mortality rate, but this disease still claims half a million lives every year. Because of their lower mortality burden, other parasitic diseases rarely make headlines. However, toxoplasmosis and cryptosporidiosis also pose important health problems. In immunocompetent hosts, toxoplasmosis is characterized by mild-flu symptoms, whereas newborns and immunocompromised patients may suffer from severe ocular infections or encephalitis. Primary infection with *Toxoplasma gondii* is also associated with fetal malformations or death of the foetus. Additionally, *Cryptosporidium parvum* has been recently identified as one of the leading causes of diarrhoeal disease in children below 2 years of age in developing countries (Kotloff *et al*., 2013). *Toxoplasma, Cryptosporidium* and several other Apicomplexa (e.g. *Theileria*, *Babesia*, and *Eimeria*) also affect livestock and are associated with important economic losses. A lack of effective anti-parasitic vaccines combined with an increase in drug resistance, rapid geographical expansion of vectors, extensive human migration and global transportation of merchandise make parasitic diseases among the most important public health challenges (Nyame *et al*., 2004). New therapies are needed and a comprehensive understanding of the molecular basis of host–pathogens interactions, together with basic parasite biology, will be crucial for the design of highly specific and efficient anti-parasitic drugs.

Glycans and glycan-binding proteins are known to play a role of paramount importance in host–pathogen interactions. Adhesion of parasites or other microbes to host cells involves interactions between glycan-binding proteins, also called lectins or adhesins, and glycan receptors. These interactions are a prerequisite for infection and often define the tropism of the pathogen. Furthermore, parasite glycans are often antigenic and may trigger both the innate and adaptive immune responses of the host. In addition, glycosylation of intracellular proteins can play roles in signalling and affect parasite proliferation and virulence. Studying the major roles of glycans in promoting parasitic infections and evading host immune responses may lead to the development of novel therapeutic agents, the identification of vaccine candidates and the development of novel diagnostic tools (Guha-Niyogi *et al*., 2001; Mendonca-Previato *et al*., 2005; Rodrigues *et al*., 2015; Goddard-Borger and Boddey, 2018).

Until the mid-1990s only scarce and often controversial information existed regarding glycosylation in apicomplexan parasites (Schwarz and Tomavo, 1993). In this pre-genomic era, glycosylphosphatidylinositols (GPIs) were shown to be synthesized by *T. gondii* and *P. falciparum*. As in other protozoan parasites, these glycans are abundantly present as proteins anchors or as free glycolipids and are essential for parasite survival (reviewed in Debierre-Grockiego and Schwarz, 2010). The availability of whole genome sequences enabled predicting biosynthetic pathways of glycans in various apicomplexa and revealed divergence between species of this phylum (Macedo *et al*., 2010; Cova *et al*., 2015; Samuelson and Robbins, 2015). The presence of a few Alg (Asparagine-linked glycosylation) genes in *Plasmodium falciparum* genome challenged the belief that *Plasmodium* does not express any *N*-glycosylated proteins. Indeed this parasite was shown to synthesize rudimentary *N*-glycans containing 1 or 2 *N*-acetylglucosamine (GlcNAc) residues, in agreement with the set of genes present in this organism (Bushkin *et al*., 2010). In contrast, *Toxoplasma* and other coccidian parasites maintained a larger *N*-glycan machinery and are able to synthesize a *N*-glycan precursor with 2 GlcNAc, 5 mannose (Man) and up to 3 glucose (Glc) residues (reviewed in Samuelson and Robbins, 2015).

In this review, we focus on other types of glycosylation recently described. *Plasmodium falciparum* is thought to be devoid of mucin-type *O*-glycans since the necessary genes and donor substrate UDP-*N*-acetylgalactosamine (UDP-GalNAc) are lacking in this parasite (Templeton *et al*., 2004; Cova *et al*., 2015; Lopez-Guttierez *et al*., 2017). Similarly, no homologue of the *O*-*N*-acetylglucosaminyltransferase (OGT), responsible for dynamic glycosylation of nuclear and cytoplasmic proteins, is present in the genome of the malaria parasite (Banerjee *et al*., 2009). In striking contrast, *Toxoplasma* adds *O*-linked GalNAc to mucin-like proteins, modifies nuclear proteins using an *O*-fucosyltransferase (OFT) similar to OGT, and glycosylates the cytosolic protein Skp1.

In the second part of this review, we address *O*-fucosylation and *C*-mannosylation of thrombospondin type 1 repeats (TSRs), newly described in key adhesins of both *Plasmodium* and *Toxoplasma*.

## Mucin-type O*-Glycans*

Transfer of *N*-acetylgalactosamine (GalNAc) to the hydroxyl group of specific serine (Ser) or threonine (Thr) residues initiates *O*-GalNAc glycosylation, a common post-translational modification of secreted or membrane-associated proteins in eukaryotes. This type of glycosylation is also referred to as mucin-type *O*-glycosylation, since mucins carry hundreds of heterogeneous *O-*GalNAc glycans in specific domains composed of Ser-, Thr-, and proline (Pro)-rich tandem repeats. This high *O*-GalNAc glycans density controls the chemical, physical and biological properties of mucins (Brockhausen and Stanley, 2017).

**Fig. 1.**
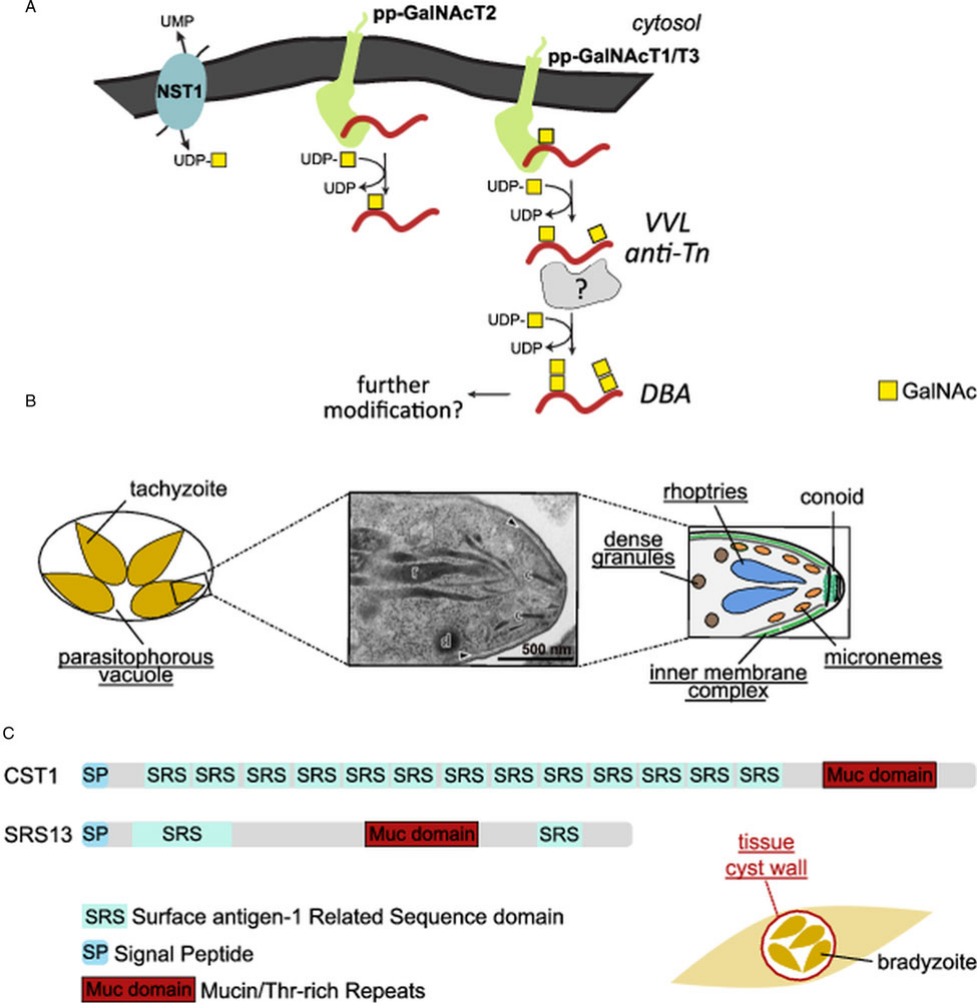
Mucin-type *O*-glycosylation in *T. gondii.* (A) Model for mucin-type glycosylation in tachyzoites and bradyzoites. Mucin domains are modified with GalNAc in a hierarchical manner by the activity of pp-GalNAcT2, followed by pp-GalNAcT1 and T3. The activity of these enzymes is dependent of the import of UDP-GalNAc in the Golgi by TgNST1. The resulting structures are recognized by *Vicia villosa* lectin (VVL) and the anti-Tn antibody. A still unknown glycosyltransferase is believed to transfer a second GalNAc residue, leading to the GalNAc*α*1,3GalNAc epitope recognized by *Dolichos Biflorus* agglutinin (DBA). (B) Candidate *O*-glycosylated proteins have been identified by lectin enrichment in tachyzoites. They localize to secretory organelles found at the apical end of the parasite (as shown by the electron micrograph and the schematic), the inner membrane complex, or the parasitophorous vacuole. Rhoptries, *r*; conoid, *c*; inner membrane complex, *dark arrow*. Subpellicular microtubules are shown in *gray* in the schematic but are not visible in the micrograph. (C) Bradyzoites are surrounded by a glycan-rich cyst wall containing the proteins CST1 and SRS13. Both proteins contain a mucin domain with Thr-rich repeats extensively modified by *O*-linked GalNAc glycans. Glycosylation of CST1 confers rigidity to the cyst wall.

Mucin-type *O*-glycosylation is initiated by a family of UDP-GalNAc: polypeptide *N*-acetylgalactosaminyltransferases (pp-GalNAcTs) evolutionary conserved from unicellular eukaryotes to mammals. They catalyse the transfer of GalNAc from the donor UDP-GalNAc to the hydroxyl group of Ser or Thr residues in acceptor proteins to form GalNAc*α*1-*O-*Ser/Thr. This structure known as the Tn antigen is usually elongated further to give rise to a variety of complex *O*-GalNAc glycans in mammalian cells (Brockhausen and Stanley, 2017). The genome of *T. gondii* encodes five putative pp-GalNAcTs (pp-GalNAcT1: TGGT1_259530; pp-GalNAcT2: TGGT1_258770; pp-GalNAcT3: TGGT1_318730; pp-GalNAcT4: TGGT1_256080; pp-GalNAcT5: TGGT1_278518) (Wojczyk *et al*., 2003; Stwora-Wojczyk *et al*., 2004*a*, 2004*b*; Tomita *et al*., 2017; Gas-Pascual *et al*., 2019). *Toxoplasma gondii* pp-GalNAcT1, T2 and T3 are constitutively expressed in both tachyzoites and bradyzoites, whereas pp-GalNAcT4 and T5 are expressed in the cat enteroepithelial stages, and T5 is additionally found in oocysts (Tomita *et al*., 2017). These enzymes have a type II topology with a short N-terminal cytoplasmic tail, a single transmembrane domain, a stem region and a conserved catalytic domain (Fig. 1A). With the exception of pp-GalNAcT4, they also have a C-terminal, ricin-like lectin domain. The catalytic domain adopts a glycosyltransferase A-fold with a DxH motif involved in divalent ion binding (Stwora-Wojczyk *et al*., 2004*b*; Tomita *et al*., 2017). As in mammals, the enzymes seem to act in a hierarchical manner. The enzyme pp-GalNAcT2 is the priming glycosyltransferase required for initial glycosylation of the mucin-like domain (Tomita *et al*., 2017). *O*-glycosylation of neighbouring acceptor sites is then carried out by the follow-on pp-GalNAcTs, pp-GalNAcT1 and T3, leading to a densely glycosylated mucin-like domain (Fig. 1A). *In vitro*, both pp-GalNAcT1 and T3 are only able to use pre-glycosylated acceptor peptides but not unglycosylated ones (Wojczyk *et al*., 2003; Stwora-Wojczyk *et al*., 2004*b*). Active pp-GalNAcTs have also been described in *Cryptosporidium* (Bhat *et al*., 2013; Haserick *et al*., 2017; DeCicco RePass *et al*., 2018), while the genome of *Plasmodium* lacks genes encoding these enzymes.

As early as the late 1970s, it was observed that *Dolichos Biflorus* agglutinin (DBA), a lectin with specificity for GalNAc*α*1,3GalNAc, effectively stained the glycosylated wall of *Toxoplasma* cysts that contain bradyzoites (slow-growing forms) (Sethi *et al*., 1977). DBA and other lectins that recognize non-reducing terminal GalNAc residues, such as *Vicia villosa* lectin (VVL), were used for affinity purification of *T. gondii* glycoproteins. Coupled with mass spectrometry (MS), this lectin-capture approach identified candidate glycoproteins, including components of the tissue cyst wall, secreted proteins from the secretory organelles (rhoptries, micronemes and dense granules), and proteins from the parasitophorous vacuole and the inner membrane complex (Luo *et al*., 2011; Wang *et al*., 2016) (Fig. 1B). Additionally, transfer of GalNAc to the hydroxyl of Ser/Thr in mucin-like domains by pp-GalNAcTs generates GalNAc*α*1-Ser/Thr, which is immunogenic in the host. Antibodies directed against this Tn antigen recognized at least 6 proteins ranging from 20 to 60 kDa (Gas-Pascual *et al*., 2019). DBA staining of the cyst wall is principally due to *O*-glycosylation of the mucin-like glycoprotein CST1, since deletion of CST1, pp-GalNAcT2, or pp-GalNAcT3 led to a loss of staining by this lectin (Tomita *et al*., 2013, 2017). CST1 confers structural rigidity to the cyst wall, and the *O*-GalNAc modification is required for this function (Tomita *et al*., 2017). Like CST1, SRS13 (surface antigen-1 related sequence 13) contains a Thr-rich mucin-like domain that is heavily *O*-glycosylated by pp-GalNAcT2 and T3 (Fig. 1C). This protein is upregulated in bradyzoites but is dispensable for cell wall formation (Tomita *et al*., 2018). In contrast *T. gondii* proteophosphoglycan 1 (*Tg*PPG1), a Ser/Pro rich-protein that shows similarities to the proteophosphoglycans of *Leishmania* parasites, enhances cell wall formation. Based on its retention in the stacking gel of SDS-PAGE, *Tg*PPG1 is likely highly glycosylated (Craver *et al*., 2010; Tomita *et al*., 2013). The prominent role of *O*-GalNAc glycosylation in the encysted form of *Toxoplasma* is highlighted by deletion of the genes encoding pp-GalNAcT2 and T3 or the nucleotide sugar transporter 1 (*Tg*NST1, TGGT1_267380), which imports UDP-GalNAc and UDP-*N*-acetylglucosamine (UDP-GlcNAc) into the endoplasmic reticulum/Golgi apparatus for biosynthesis of glycans. Deficiency of pp-GalNAcT2 or T3 leads to fragile brain cysts, likely due to the absence of CST1 glycosylation, while a lower cyst load in the brain was observed in *Tg*NST1-deficient parasites. In contrast, deletion of these genes did not significantly impact the tachyzoite stage of the parasite (Caffaro *et al*., 2013; Tomita *et al*., 2017).

Recently, a study of total *O*-glycans released by *β*-elimination from *Toxoplasma* tachyzoites suggested that this parasite stage expresses only one major mucin-type *O*-glycan containing two *N*-acetylhexosamines (Gas-Pascual *et al*., 2019). The epimerase generating UDP-GalNAc from UDP-GlcNAc (GalE) is necessary for the formation of this *O*-linked disaccharide. This result indicates the presence of at least one GalNAc residue at the reducing end, which is consistent with the expression of pp-GalNAcTs in this parasite stage. Based on previous studies with lectins (Wang *et al*., 2016; Tomita *et al*., 2017), the authors suggest the structure GalNAc-GalNAc and possibly GalNAc*α*1,3GalNAc*α*1-Ser/Thr, which is preferentially recognized by DBA (Gas-Pascual *et al*., 2019). The glycosyltransferase that extends the *O*-linked GalNAc has not yet been identified (Fig. 1A).

## Nucleocytosolic glycosylation in *T. gondii*

Studies performed in the last 30 years have underlined the fact that glycosylation of proteins in the nucleus and cytosol is not an exception, but a conserved feature in most eukaryotes. *O*-GlcNAcylation, the modification of nucleocytoplasmic proteins with a single GlcNAc residue, was first described in mammals in the early 80s. It was followed a decade later by the initial identification of the Skp1 glycosylation pathway in *Dictyostelium discoideum* (West and Hart, 2017) and, in the last 2 years, the identification of *O*-mannose in yeast (Halim *et al*., 2015) and *O*-fucose in *T. gondii* and *Arabidopsis thaliana* (Bandini *et al*., 2016; Zentella *et al*., 2017).

### The Skp1 glycosylation pathway

**Fig. 2.**
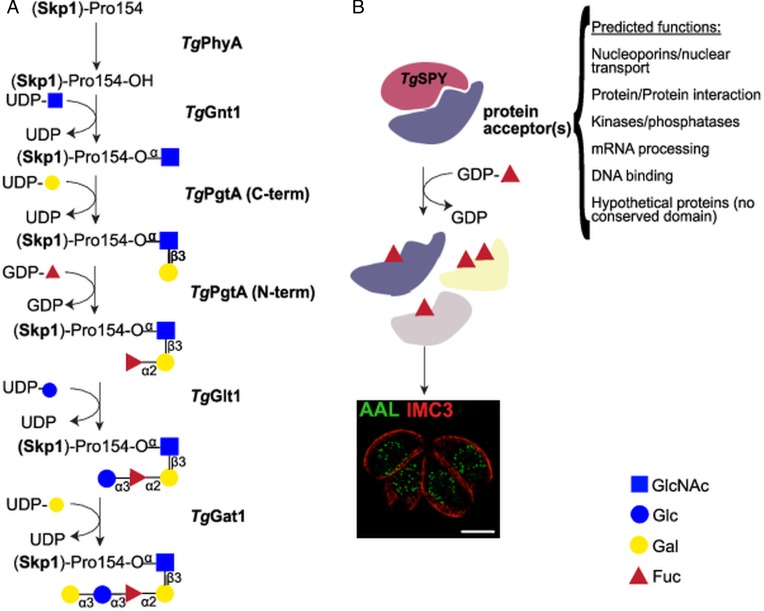
Nucleocytosolic glycosylation pathways in *T. gondii.* (A) Skp1 glycosylation pathway. Proline 154 of the Skp1 protein is first hydroxylated by *Tg*PhyA and then modified by a pentasaccharide of the composition Gal*α*1,3Glc*α*1,3Fuc*α*1,2Gal*β*1,3GlcNAc*α*1– which is assembled by four glycosyltransferases. Transfer of *α*GlcNAc to the hydroxylated proline by *Tg*Gnt1 is followed by the sequential transfer of *β*1,3-linked Gal and *α*1,2Fuc by the bifunctional enzyme *Tg*PgtA. *Tg*Glt1 and *Tg*Gat1 transfer the remaining two sugars, Glc and Gal, both in *α*1,3 linkage. (B) Nucleocytosolic *O*-fucosylation. *Tg*SPY, a paralogue of animal *O*-GlcNAc transferases, modifies more than 60 proteins with one or more *O*-linked fucose residues. Structured illumination microscopy of tachyzoites suggests that the *O*-fucosylated proteins form assemblies that localize at the nuclear periphery. AAL: *Aleuria aurantia* lectin (binds to fucose); IMC3: marker for *T. gondii* inner membrane complex.

In *T. gondii*, the proline 154 of Skp1 (S-phase kinase-associated protein 1), an adaptor of Skp1/Cullin1/F-box protein (SCF)-class E3 ubiquitin ligases, is hydroxylated and further modified by a pentasaccharide (Rahman *et al*., 2016, 2017; West and Hart, 2017; Gas-Pascual *et al*., 2019) (Fig. 2A). This glycosylation pathway is involved in the response of *Dictyostelium* to changes in environmental oxygen (West *et al*., 2010). Detection and monitoring of oxygen levels is a function required by all cells and, in eukaryotes, cytosolic proline 4-hydroxylases (P4Hs) act as key oxygen sensors (Xu *et al*., 2012*a*). While the hydroxylation mechanism is conserved between animals and protists, the protein targets differ. In animals, P4Hs modify the transcriptional co-factor, hypoxia-inducible factor-*α* (HIF*α*). At normal oxygen levels, hydroxylation of HIF*α* leads to its poly-ubiquitination and subsequent proteosomal degradation, blocking the transcription of hypoxia-specific genes (Xu *et al*., 2012*b*). In protists such as *Toxoplasma* and *Dictyostelium* a single proline on Skp1 is hydroxylated and then further modified by a pentasaccharide. This modification does not lead to Skp1 degradation, but instead likely influences poly-ubiquitination and targeting to the proteasome of many other proteins (Xu *et al*., 2012*a*). In *T. gondii*, gene disruption of either the proline hydroxylase (*phyA*) or the four glycosyltransferases required for the pentasaccharide synthesis, although tolerated, leads to defects in parasite replication in the host. As might be expected, the strongest phenotype is observed in mutants lacking glycosylation, while milder growth defects are observed in mutants with reduced glycosylation (Xu *et al*., 2012*a*; Rahman *et al*., 2016, 2017).

*Toxoplasma gondii* pentasaccharide was defined as Gal*α*1,3Glc*α*1,3Fuc*α*1,2Gal*β*1,3GlcNAc*α*1- (Gal, Galactose; Glc, Glucose; Fuc, Fucose) based on glycopeptide MS/MS data, NMR studies, and homology to the *Dictyostelium* biosynthetic pathway (Rahman *et al*., 2016, 2017; West and Hart, 2017). After hydroxylation of proline 154 by *Tg*PhyA (TGGT1_232960) (Xu *et al*., 2012*b*), the first glycosyltransferase, *Tg*Gnt1 (TGGT1_315885), transfers GlcNAc in an *α* linkage (Rahman *et al*., 2016), resulting in an alkali-resistant glycosylation (Fig. 2A). Gnt1 belongs to the Carbohydrate-Active Enzyme (CAZy) glycosyltransferase (GT) 60 family (or GT60), a group that includes also *Trypanosoma cruzi* UDP-GlcNAc: polypeptide *α*-*N*-acetylglucosaminyltransferase (Heise *et al*., 2009; Lombard *et al*., 2014). Incubation of *Dd*Skp1 with UDP-[^3^H]GlcNAc and cytosolic extracts of wild type *T. gondii,* but not of Δ*gnt1* parasites, resulted in the transfer of [^3^H]-GlcNAc to *Dd*Skp1, consistent with the identification of *Tg*Gnt1 as the UDP-GlcNAc:HyPro Skp1 polypeptide *α*-*N*-acetylglucosaminyltransferase. This activity is dependent on the presence of PhyA, indicating the specificity of Gnt1 for the hydroxylated proline as acceptor (West *et al*., 2006; Rahman *et al*., 2016).

Transfer of the second and third sugars is catalysed by *Tg*PgtA (TTGT1_260650), a bifunctional *β*1,3-galactosyltransferase (*β*1,3-GalT)/*α*1,2-fucosyltransferase (*α*1,2-FucT) (Fig. 2A). PgtA is organized in two separate domains belonging to the glycosyltransferase family GT74 and GT2, each responsible for one specific glycosyltransferase activity. The GT74 family was founded with *D. discoideum* PgtA and contains several eukaryotic and bacterial *α*1,2-FucTs (West *et al*., 2010). The GT2 domain of PgtA mediates the transfer of *β*1,3Gal (Rahman *et al*., 2016) and belongs to a very large family of functionally diverse inverting GTs. *Tg*PgtA presents a GT74-GT2 tandem organization, while the domains are swapped in *Dictyostelium* PgtA (Van Der Wel *et al*., 2002). The same assay setup described above for *Tg*Gnt1 was used to demonstrate both FucT and GalT activities from *T. gondii* cytosolic extracts. Transfer of Fuc required the presence of UDP-Gal in the reaction, but not vice versa, indicating that Gal and Fuc are transferred sequentially to GlcNAc*α*-O-Skp1 (Rahman *et al*., 2016).

*Dictyostelium discoideum* and *T. gondii* Skp1 glycosylation differ in the nature of the two distal sugars. In *Dictyostelium*, a single glycosyltransferase *Dd*AgtA transfers two *α*1,3Gal residues (Ercan *et al*., 2006; Schafer *et al*., 2014; Sheikh *et al*., 2017), while the core trisaccharide of *T. gondii* Skp1 is sequentially modified with an *α*1,3Glc and an *α*1,3Gal added by *Tg*Glt1 and *Tg*Gat1, respectively (Rahman *et al*., 2017; Gas-Pascual *et al*., 2019) (Fig. 2A). *Tg*Glt1 (TGGT1_205060), a member of the GT32 family, was identified as a UDP-Glc: fucoside *α*1,3-glucosyltransferase using multiple approaches. Liquid chromatography-tandem mass spectrometry (LC-MS/MS) analysis of immunoprecipitated Skp1 in parasites deficient for *glt1* revealed the accumulation of protein modified by the trisaccharide only. Moreover, 1D and 2D NMR analysis of the product obtained with recombinant *Tg*Glt1 showed that the enzyme transferred Glc in an *α*1,3 linkage (Rahman *et al*., 2017). Finally, *Tg*Gat1 (TGGT1_310400), the last enzyme involved in the pathway, has not yet been extensively characterized. This enzyme is a member of the GT8 family, which comprises retaining UDP-sugar transferases, including glycogenins (Lombard *et al*., 2014; West and Hart, 2017) and a knock-out cell line has just been reported (Gas-Pascual *et al*., 2019).

Recent studies in *D. discoideum* have shed some light on the role of Skp1 glycosylation. Structural and molecular dynamics studies suggested that the pentasaccharide stabilizes the flexible F-box-binding domain on Skp1, favouring an open conformation that improves binding to its ubiquitination partners, e.g. Cullin1 (Sheikh *et al*., 2017). While key Skp1 amino acids involved in interacting with the pentasaccharide are conserved in *T. gondii* Skp1, further studies are necessary to understand if the *Dictyostelium* model extends to this parasite. Furthermore, unlike *Dictyostelium* that during its development moves between environments with very different oxygen levels, *T. gondii* spends most of its life cycle in low oxygen (intracellular stages) or anoxia (oocysts released in the intestine) settings (Xu *et al*., 2012*a*). If this difference is relevant only at the level of post-translational modification kinetics or has a wider importance in the function of this signalling pathway remains to be studied.

### *O*-fucosylation of nucleocytosolic proteins in *T. gondii*

Immunofluorescence and enrichment using the fucose-specific *Aleuria aurantia* lectin (AAL) led to the identification at least 69 *O*-fucosylated proteins that localize to the nuclear periphery of *T. gondii* tachyzoites (Bandini *et al*., 2016) (Fig. 2B). Glycopeptide analysis identified amino acids sequences modified with up to 6 deoxyhexoses (dHex) linked to a Ser or Thr residue, and gas chromatography-MS (GC-MS) compositional analysis after reductive *β*-elimination identified the dHex as Fuc. This was further confirmed by the loss of AAL binding after transient gene disruption of GDP-mannose 4,6-dehydratase, a key enzyme in GDP-Fucose (GDP-Fuc) biosynthesis (Bandini *et al*., 2016). While no consensus *O*-fucosylation motif could be identified, the observed peptides are rich in Ser, Thr and non-polar amino acids. In addition, about a third of the identified peptides contain long homoserine repeats.

The identified *O*-fucosylated proteins have a variety of predicted functions, from nuclear transport and mRNA processing to signalling and protein–protein interactions (Bandini *et al*., 2016) (Fig. 2B). These protein families and modified peptides are highly reminiscent of the sequences modified by the *O*-GlcNAc transferase (OGT) in animals (Bond and Hanover, 2015). Indeed, the *T. gondii* genome encodes for a putative OGT (TGGT1_273500), having high homology to SPY-like enzymes (Banerjee *et al*., 2009; Olszewski *et al*., 2010). The SPY-like *vs* SEC-like classification originates in plants, which were long thought to encode two OGTs: SPINDLY (SPY) and SECRET AGENT (SEC) (Olszewski *et al*., 2010). SPY-like and SEC-like enzymes are both characterized by an N-terminal domain composed of tetratricopeptide repeats and a C-terminal CAZy GT41 catalytic domain (Olszewski *et al*., 2010; Lombard *et al*., 2014). They differ in the number of tetratricopeptide repeats and the sequence of the GT41 domain (Olszewski *et al*., 2010), even though many catalytic residues are conserved (Zentella *et al*., 2017). Animals and fungi OGTs classify as SEC-like and are involved in *O*-GlcNAc transfer just as *A. thaliana* SEC (Hartweck *et al*., 2002). In contrast, *At*SPY has recently been shown to be an *O*-fucosyltransferase (Zentella *et al*., 2017) and not an OGT. Consistent with this report, knock-out of *spy* in *T. gondii* resulted in the loss of AAL binding, strongly suggesting the nucleocytosolic *O*-fucosyltransferase activity of *Tg*SPY. *Spy*-deficient parasites were viable but displayed a mild defect in replication in host cells *in vitro* (Gas-Pascual *et al*., 2019). Structured illumination microscopy (SIM) showed that AAL binds in a punctate pattern to the nuclear periphery of *T. gondii* tachyzoites, suggesting that the *O*-fucosylated proteins are forming assemblies (Fig. 2B). Furthermore a Ser-rich domain fused to YFP is *O*-fucosylated when expressed in tachyzoites and localizes to the nuclear periphery (Bandini *et al*., 2016). Co-labelling with a Phenylalanine-Glycine (FG)-repeat nucleoporin suggests that these assemblies are found in close proximity to the nuclear pore complex and four out of the six *T. gondii* FG-repeat nucleoporins are found in the AAL-enriched fraction (Bandini *et al*., 2016; Courjol *et al*., 2017). This pattern was also observed in bradyzoites and sporozoites, but not in oocysts, suggesting that nuclear *O*-fucosylation might be regulated during the parasite life cycle (Fig. 4).

Nuclear staining with AAL was observed not only in *Hammondia hammondi* and *Neospora caninum*, the two species most closely related to *T. gondii*, but also *Cryptosporidum parvum* sporozoites (Bandini *et al*., 2016), which are all predicted to encode a SPINDLY orthologue. Interestingly, nucleocytosolic extracts of *T. gondii* or *C. parvum* as well as the recombinant *C. parvum* SPY enzyme have been shown to have OGT activity *in vitro* (Banerjee *et al*., 2009; Perez-Cervera *et al*., 2011). Recent work has also reported the presence of *O*-GlcNAcylated proteins both in *P. falciparum* and *T. gondii*, using enrichment of proteins with terminal GlcNAc and identification by MS/MS (Kupferschmid *et al*., 2017; Aquino-Gil *et al*., 2018). Unfortunately, the peptide fragmentation technique used in these studies did not allow observation of glycopeptides. Future work will likely address the substrate specificity of SPY protein and its potential implication in *O*-GlcNAcylation. Work published in *A. thaliana* so far describes only one protein acceptor for SPY, the master regulator DELLA, which is also a substrate for SEC (Zentella *et al*., 2016, 2017). However, no subcellular localization studies have been performed in this system. Further studies will define if *O*-fucosylation directs proteins at the nuclear periphery and if this role is conserved in other eukaryotic lineages.

## Glycosylation of thrombospondin type 1 repeats

In 2015, Cova and colleagues pointed out the conservation of enzymes for GDP-mannose (GDP-Man) and GDP-Fuc biosynthesis in the genome of different apicomplexan parasites (Cova *et al*., 2015). These nucleotides sugars, required for glycosylation reactions, were also shown to be present in *Plasmodium falciparum* blood stages by LC-MS/MS (Sanz *et al*., 2013). A substantial pool of GDP-Man was expected since Apicomplexa synthesize abundant GPI-anchors, but the presence of GDP-Fuc was more surprising considering that no fucose-containing glycoconjugates had been identified. The existence of fucosylated proteins in the nucleus and the Skp1 pathway mentioned above would explain the requirement for GDP-Fuc in *T. gondii* and a few other Apicomplexa, but not in *Plasmodium* spp. However, several Apicomplexa adhesins have thrombospondin type-1 repeats (TSRs), and homologs of the enzymes required for *O*-fucosylation and *C*-mannosylation of TSRs in higher eukaryotes can be identified in this phylum (Buettner *et al*., 2013; Sanz *et al*., 2013; Cova *et al*., 2015).

**Fig. 3.**
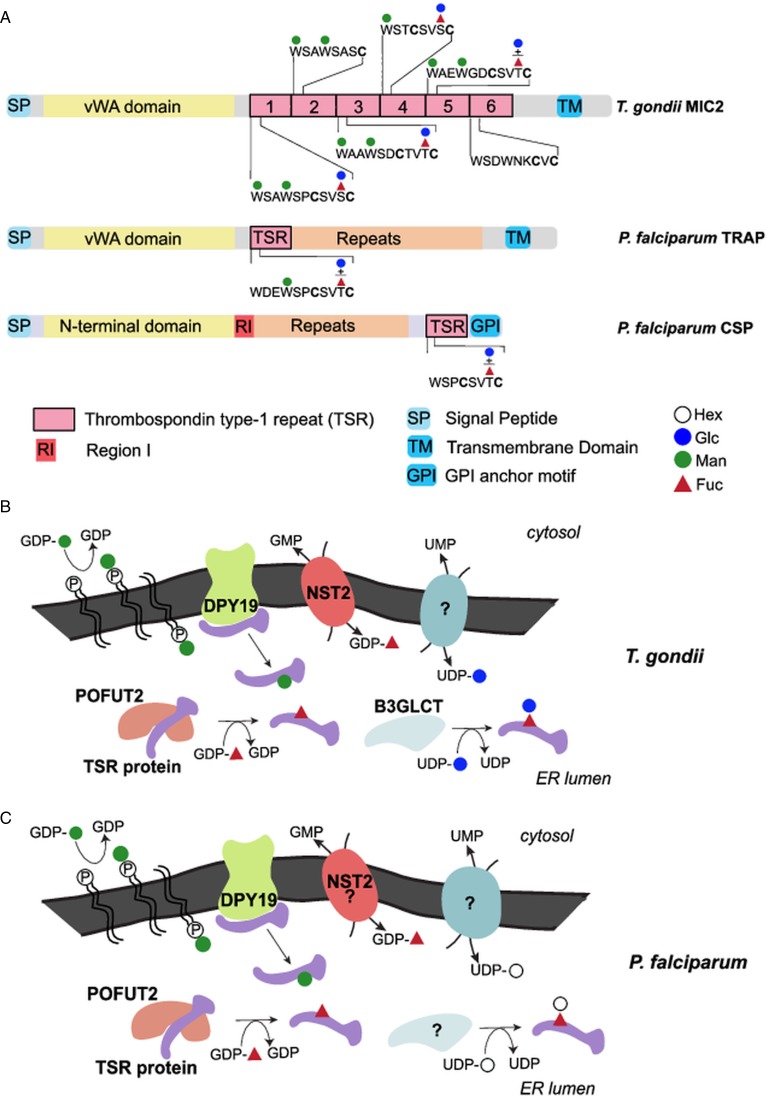
*O*-fucosylation and *C*-mannosylation on TSR repeats. (A) Summary of the mass spectrometry evidence for TSR glycosylation in the two parasites. The presence of a plus sign between Glc and Fuc indicates that glycopeptides were observed for two different glycoforms: only dHex (Fuc) or Hex-dHex (FucGlc). (B) Schematic representation of the two TSR glycosylation pathways in *T. gondii*. DPY19 transfers Man from dolichol-phosphate-mannose to tryptophan (W) residues on TSRs. POFUT2, a soluble protein in most eukaryotes, modifies Ser/Thr in the CX_2−3_S/TCX_2_G motif with Fuc, which can be further elongated by addition of Glc by B3GLCT. This glycosylation requires the GDP-Fuc transporter NST2 and a UDP-Glc transporter. (C) In *P. falciparum*, POFUT2 and DPY19 are known to modify TSRs with *O*-Fuc and *C*-Man, as detailed for *Toxoplasma*. The identities of the B3GLCT and the Hex transferred on Fuc have not yet been ascertained. NST2 is the predicted GDP-Fuc transporter.

TSRs are ancient protein modules that emerged before the separation of nematodes and chordates (Hutter *et al*., 2000). These domains contain approximately 60 amino acids and are typically organized as an elongated three stranded *β*-sheet, with three conserved disulfide bridges and stacked tryptophan and arginine residues that stabilize the TSR structure (Tan *et al*., 2002). Present in several proteins in vertebrates, these adhesive domains play roles in immunity, adhesion, neuronal development and signalling (Adams and Tucker, 2000; Shcherbakova *et al*., 2017). In Apicomplexa, the extracellular domains of several adhesins, including *Plasmodium* thrombospondin-relative anonymous protein (TRAP, PF3D7_1335900), circumsporozoite protein (CSP, PF3D7_0304600), and *T. gondii* micronemal proteins 2 (MIC2, TGME49_201780) contain at least one TSR domain (Tucker, 2004; Carruthers and Tomley, 2008) (Fig. 3A). These adhesins interact with receptors present at the host cell surface and are linked *via* their cytoplasmic tail to the glideosome, a molecular machine necessary for parasite motility and host cell invasion (Frenal *et al*., 2017). *Plasmodium* spp. express several TRAP variants that mediate motility, invasion, and egress at different stages of the parasite life cycle, both in the mammalian host and in the mosquito (Sultan *et al*., 1997; Dessens *et al*., 1999; Wengelnik *et al*., 1999; Combe *et al*., 2009; Steinbuechel and Matuschewski, 2009; Bargieri *et al*., 2016). In *T. gondii*, MIC2 plays a comparable role in tachyzoites (Huynh *et al*., 2003; Huynh and Carruthers, 2006; Gras *et al*., 2017). In addition, several Apicomplexa TSR-containing proteins are uncharacterized, and their functions remain unknown.

### *O*-fucosylation

Besides database mining, the presence of *α*-*O*-fucosylation in Apicomplexa was strongly suggested by MS/MS analyses of TRAP and CSP from *P. falciparum* sporozoites, which demonstrated modification of the TSRs by an *O*-linked hexose-deoxyhexose (Hex-dHex) (Swearingen *et al*., 2016) (Fig. 3A). This disaccharide was assumed to be Glc*β*1,3Fuc-*α*-*O*-Ser/Thr as described in mammalian TSRs (Kozma *et al*., 2006; Luo *et al*., 2006; Vasudevan and Haltiwanger, 2014). A clear homolog of the protein *O*-fucosyltransferase 2 (POFUT2) could indeed be identified in the genome of apicomplexan parasites (Cova *et al*., 2015). POFUT2 catalyses *α*-linked fucosylation of Ser or Thr residues in the consensus sequence C^1^X_2−3_S/TC^2^X_2_G, where C^1^ and C^2^ are the first two conserved cysteines in the TSR (Luo *et al*., 2006). The ability of recombinant *Plasmodium vivax* POFUT2 (PVX_098900) to use GDP-Fuc as donor substrate and to modify the CX_2−3_S/TCX_2_G motif of TRAP and CSP was later demonstrated using an *in vitro* glycosyltransferase assay (Lopaticki *et al*., 2017). In *T. gondii*, MIC2 is intensely stained with an antibody recognizing the Glc*β*1,3Fuc epitope, and four TSRs of MIC2 that contain an *O*-fucosylation motif (TSR1, 3, 4 and 5) are modified by a Hex-dHex disaccharide by MS/MS analysis of glycopeptides (Fig. 3A). This modification was abolished by deletion of the gene encoding POFUT2 (TGME49_273550) thereby proving the function of this glycosyltransferase in *T. gondii* (Fig. 3B) (Bandini *et al*., 2019; Gas-Pascual *et al*., 2019; Khurana *et al*., 2019).

Deletion of the gene encoding POFUT2 in *P. falciparum* (PF3D7_0909200) and, in at least one report, in *T. gondii* indicates that, in apicomplexa as in mammals, *O*-fucosylation plays a role in the stabilization and trafficking of TSR-containing proteins (Lopaticki *et al*., 2017; Bandini *et al*., 2019). As in mammals, the loss of *α*-*O*-fucosylation affected proteins differently. In *Plasmodium* sporozoites, the cellular level of TRAP was significantly decreased, whereas CSP was not affected (Lopaticki *et al*., 2017). Consistent with the importance of TRAP in adhesion and motility, hepatocyte invasion by sporozoites was impaired leading to a reduced liver parasite load. Loss of *O*-fucosylation also impacted on infection of mosquito midgut epithelial cells by ookinetes (Lopaticki *et al*., 2017). This phenotype could be related to a destabilization of the circumsporozoite and TRAP-related protein (CTRP, PF3D7_0315200), since this protein is involved in the motility of ookinetes and contains *O*-fucosylation motifs (Dessens *et al*., 1999; Lopaticki *et al*., 2017). However, several other proteins contain the CX_2_S/TCX_2_G motif and might contribute to the phenotype observed in POFUT2-deficient parasites. In blood stage parasites, POFUT2 is expressed but dispensable for growth (Sanz *et al*., 2013; Lopaticki *et al*., 2017). Similarly, no prominent effects were observed in the blood stages of *P. falciparum* and *P. berghei* mutants lacking enzymes involved in GDP-Fuc biosynthesis (Gomes *et al*., 2015; Sanz *et al*., 2016). To date, no *O*-fucosylated protein has been identified in blood stages. The TSR-containing TRAP variants MTRAP (merozoite TRAP), SPATR (sporozoite protein with an altered thrombospondin repeat) and PTRAMP (*Plasmodium* thrombospondin-related apical merozoite protein) are devoid of the canonical POFUT2 consensus motif, suggesting that they are not *O*-fucosylated (Lopaticki *et al*., 2017).

As in *Plasmodium*, deletion of *T. gondii* POFUT2 affects the trafficking and cellular levels of the major parasite adhesin MIC2. Using *in vitro* assays, the decreased adhesion and impaired ability to invade displayed by POFUT2-deficient parasites were comparable to those observed in a mutant completely devoid of MIC2 (Gras *et al*., 2017; Bandini *et al*., 2019). However, the defects in both egress and parasite replication were much less pronounced in the *O*-fucosylation mutant than in the parasites lacking MIC2. Add back of *pofut2* rescued the attachment/invasion phenotype of Δ*pofut2* parasites and restored cellular MIC2 levels, ruling out off-target mutations being the cause of the phenotype in the knockout. An identical phenotype was obtained by deletion of the nucleotide sugar transporter 2 (NST2, TGGT1_267730) establishing the specificity of this transporter for GDP-Fuc (Bandini *et al*., 2019) (Fig. 3B). *Toxoplasma gondii pofut2* was knocked out in two additional studies (Gas-Pascual *et al*., 2019; Khurana *et al*., 2019). Gas-Pascual *et al*., reported a defect in parasite replication upon its disruption, but did not address invasion or attachment as it was beyond the scope of the screen performed in this study. Finally, in Khurana *et al*., knock-out of *pofut2* had no effect on cellular levels of MIC2, parasite proliferation, or its ability to attach and invade host cells. Discrepant phenotypes between the two reported *pofut2* knockouts likely results from the different methods used for generating these cell lines and/or for assaying phenotype (Bandini *et al*., 2019; Khurana *et al*., 2019).

In mammals, *O*-linked fucose on TSRs is typically extended by a *β*1,3-linked glucose transferred by the glucosyltransferase B3GLCT, which is also involved in protein quality control (Kozma *et al*., 2006; Luo *et al*., 2006). A recent study indicates that a small percentage of TRAP and CSP from *P. falciparum* salivary glands sporozoites is modified with a Hex-dHex whereas only a dHex is found on these proteins in *P. vivax* and *P. yoelii* sporozoites (Swearingen *et al*., 2019). This glucosyltransferase belongs to the large GT31 family, which contains enzymes with various functions (Lombard *et al*., 2014). In *P. falciparum*, the parasite-infected erythrocyte surface protein 1 (PIESP1, PF3D7_0310400) shares 31% protein identity with the human B3GLCT and has been proposed as candidate B3GLCT (Swearingen *et al*., 2016). Surprisingly, this protein was localized at the surface of infected red blood cells (Florens *et al*., 2004), whereas B3GLCT would be expected to localize in the endoplasmic reticulum (ER) with POFUT2 (Lopaticki *et al*., 2017). Moreover, by saturation mutagenesis, the PF3D7_0310400 gene is predicted to be essential in asexual blood stages whereas *pofut2* is dispensable (Zhang *et al*., 2018). These data suggest that PIESP1 is not responsible for glucosylation of *O*-fucose. In *T. gondii*, however, Gas-Pascual and colleagues recently reported loss of an *O*-linked Hex-dHex disaccharide upon knock-out of the putative *b3gltc* (TGGT1_239752) indicating that this enzyme is likely responsible for elongating *O*-Fuc (Fig. 3B). A defect in the lytic cycle characterised by small lytic plaques was also reported in this mutant (Gas-Pascual *et al*., 2019). While binding of MIC2 by a Glc*β*1,3Fuc-specific antibody suggests that at least in *T. gondii* the hexose modifying *O*-Fuc is indeed Glc (Bandini *et al*., 2019), further investigations are necessary to confirm this result and the identity of the hexose in other Apicomplexa.

Nucleotide sugars, required for the biosynthesis of glycans, are mostly synthesized in the cytosol and actively transported into the ER or Golgi by the action of nucleotide sugar /nucleoside monophosphate antiporters (NSTs). The functional identification of *Tg*NST2, the GDP-fucose transporter in *T. gondii*, has been mentioned above and *P. falciparum* genome also encodes a putative GDP-sugar transporter, PF3D7_0212000 (Fig. 3C). Similarly, B3GLCT activity requires transport of the UDP-glucose donor into the ER. Bioinformatics searches, using either yeast or human NSTs as templates, identified *T. gondii* NST3 (TGGT1_254580) and *Pf*UGT (PF3D7_1113300) as putative UDP-Glc/UDP-Gal transporters because of their close homology with the yeast HUT1 transporter (Banerjee *et al*., 2008; Caffaro *et al*., 2013). In *Plasmodium* erythrocyte stages, *Pf*UGT has been localized to the ER and identified as a multidrug resistance gene (Lim *et al*., 2016). Using a CRISPR screen, the phenotype score associated with the *Tg*NST3 encoding gene (−4.9) was significantly lower than the scores associated with *pofut*2 (−0.34) and *b3glct* (−1.36) suggesting that deletion of *nst3* would have much more severe effects than the loss of *O*-fucosylation (Sidik *et al*., 2014). Similarly, *piggyBac* transposon insertional mutagenesis suggests that the gene encoding the putative UDP-Glc/UDP-Gal transporter *Pf*UGT is not mutable in asexual blood stages whereas, as mentioned above, *O*-fucosylation does not seem to play important roles in these parasite stages (Lopaticki *et al*., 2017; Zhang *et al*., 2018). These results are difficult to conciliate and suggest that *Tg*NST3 and *Pf*UGT have a different or broader substrate specificity. In metazoans, the function of the HUT1 transporter is also still unclear. Recently, the human orthologue called solute carrier 35B1 (SLC35B1), has been proposed to act as ATP/ADP antiporter (Klein *et al*., 2018).

### *C*-mannosylation

**Fig. 4.**
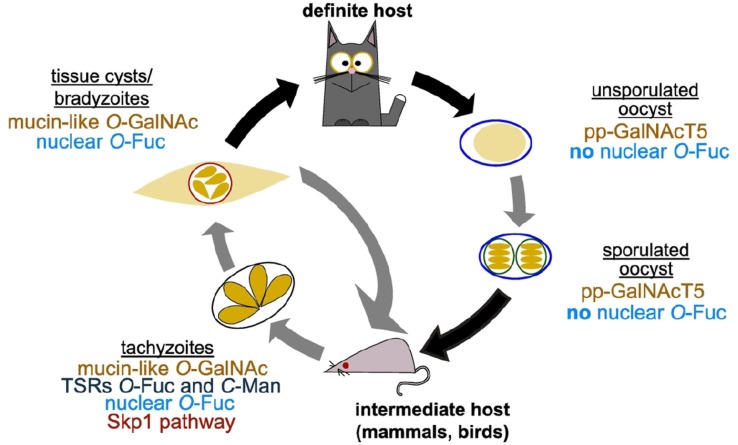
*O*- and *C*-glycosylation pathways in *Toxoplasma gondii* life cycle. *T. gondii* replicates asexually in the intermediate host with tachyzoites as the fast replicative form and bradyzoites in tissue cysts characterizing the chronic stage of infection. In felids, its definite host, *T. gondii* goes through a sexual cycle that concludes with the shedding of unsporulated oocysts that then sporulate in the environment. As shown in the schematic, all the glycosylation pathways reviewed here have been shown to be present in tachyzoites. Transfer of *O*-GalNAc to mucin-like domains is an important post-translational modification in tissue cyst wall proteins and pp-GalNAcT5 is expressed in oocysts. Nuclear *O*-fucosylation has been shown to be present also in bradyzoites and sporozoites, but is absent from oocysts.

**Fig. 5.**
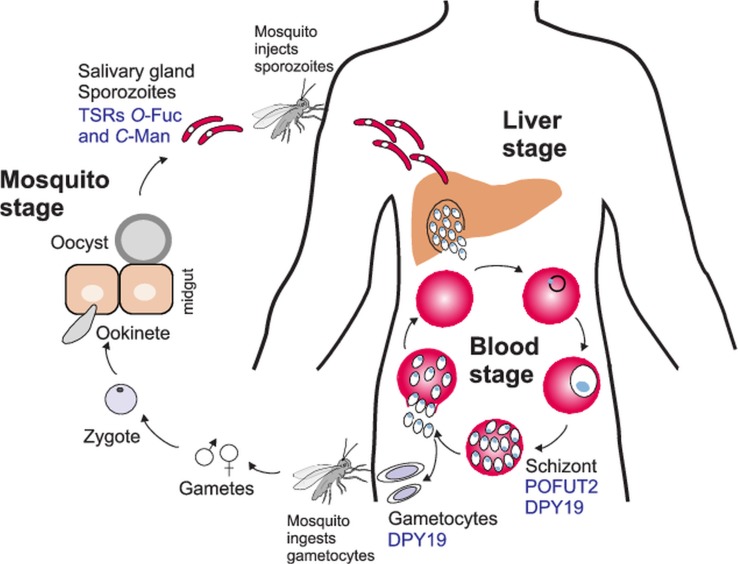
*O*- and *C*-glycosylation pathways in *Plasmodium falciparum* life cycle. During a mosquito blood meal, sporozoites are injected in the bloodstream and infect the liver. After asexual replication in hepatocytes, the parasites are released into the bloodstream where they replicate in erythrocytes to give the characteristic fever symptoms. A fraction of the parasites will develop into gametocytes that can be transmitted to the mosquito during a blood meal. After zygote formation, *Plasmodium* ookinetes infect the mosquito midgut and develop into oocysts. Sporozoites are released from the oocysts and travel to the salivary gland ready for a new infection cycle. *O*-fucosylation and *C*-mannosylation of TSRs have been demonstrated in sporozoites, but POFUT2 and DPY-19 have been detected in the asexual blood stages. DPY19 is also present in gametocytes. Studies on GDP-Fuc biosynthesis have been performed in the intraerythrocytic stages.

*C-*mannosylation is a less known protein modification, mediated by enzymes of the DPY19 family, which, in metazoans, transfer an *α*-mannose residue from dolichol-phosphate-mannose to a tryptophan (Trp) residue located in a WX_2_W or WX_2_C motif (Buettner *et al*., 2013; Niwa *et al*., 2016; Shcherbakova *et al*., 2017). As in the case of *α*-*O*-fucosylation, analysis of Apicomplexa genomes strongly suggested the existence of this modification, and MS/MS analyses confirmed *C*-hexosylation of tryptophan residues in the TSRs of *Plasmodium* TRAP and *Toxoplasma* MIC2, in sporozoites and tachyzoites, respectively (Figs 3A, 4 and 5) (Swearingen *et al*., 2016, 2019; Bandini *et al*., 2019; Khurana *et al*., 2019). In metazoans, this glycosylation is typically found on TSRs carrying a WX_2_WX_2_C^1^ sequence (that directly precedes the *O*-fucosylation site), as well as type I cytokine receptors characterized by a WSXWS signature (Hofsteenge *et al*., 1999; Julenius, 2007). However, the latter is not found in Apicomplexa. In mammals, two different enzymes (DPY19L1 and DPY19L3) are required for *C*-mannosylation of both Trp residues in the sequence WX_2_WX_2_C, whereas Apicomplexa genomes contain a single DPY19 homolog (Buettner *et al*., 2013; Shcherbakova *et al*., 2017). An *in vitro* assay using recombinant *T. gondii* (TGME49_080400) and *P. falciparum* DPY19 (PF3D7_0806200) indicated that the apicomplexan enzymes are unable to act on a WX_2_W peptide as their mammalian counterparts but modified WX_2_WX_2_C motif, suggesting that they are tailored for TSR modification. Detailed MS/MS analyses of MIC2 further indicated that both tryptophans of the WX_2_WX_2_C sequence can be modified (Fig. 3A). Finally, *in vitro* assays also showed that both *T. gondii* and *P. falciparum* DPY19 use dolichol-phosphate-mannose as donor substrate, proving their mannosyltransferase activity (Hoppe *et al*., 2018).

In mammals, *C*-mannosylation is suggested to play an important role in stabilization and/or folding of some but not all proteins (Munte *et al*., 2008; Wang *et al*., 2009; Buettner *et al*., 2013; Sasazawa *et al*., 2015; Siupka *et al*., 2015; Fujiwara *et al*., 2016; Niwa *et al*., 2016; Okamoto *et al*., 2017; Shcherbakova *et al*., 2017). Using a CRISPR/Cas9 genetic screen or a *piggyBac* transposon insertional mutagenesis, DPY19 was predicted to confer fitness to *T. gondii* tachyzoites or *P. falciparum* asexual stages, respectively (Sidik *et al*., 2014; Zhang *et al*., 2018). Further studies are required to define the function of this type of protein glycosylation in apicomplexa.

### Concluding remarks

The adaptation of Apicomplexa to parasitic lifestyle was accompanied by a reductive genome evolution involving lineage-specific gene loss (Templeton *et al*., 2004). This has led to divergent protein glycosylation in the various Apicomplexa genera. Biosynthesis of glycosylphosphatidylinositols is conserved throughout the eukaryotes and these are particularly abundant at the surface of Apicomplexa. In contrast, biosynthetic pathways involved in surface protein *N*- and *O*-glycosylation have been considerably reduced or even eliminated. Due to secondary loss of *alg* genes, *Plasmodium* expresses rudimentary *N*-glycans composed of only one or two *N*-acetylglucosamine residues, whereas *Toxoplasma N*-glycans are more elaborated (Bushkin *et al*., 2010; Samuelson and Robbins, 2015).

The synthesis of mucin-type *O*-glycosylation represents another important divergence between *Plasmodium* and *Toxoplasma*. Recent genetic and biochemical work clearly established the existence of mucin-type glycosylation in *Toxoplasma* and its importance for the rigidity of the cyst wall and parasite persistence. These *O*-GalNAc glycans likely consist of a disaccharide and are thus less complex than in other eukaryotes. Conversely, *Plasmodium* apparently lacks the enzymes involved in this protein post-translational modification.

Additionally, two distinct cytoplasmic and nuclear glycosylation pathways were recently described in *Toxoplasma*. The ubiquitin ligase adaptor Skp1 was shown to be hydroxylated and modified by a pentasaccharide; a post-translation modification required for optimal oxygen-dependent development. Lastly, more than 60 *Toxoplasma* nuclear proteins were shown to be substituted by one or more fucose residues. This modification carried out by the SPY enzyme is reminiscent of protein *O*-GlcNAcylation and might have a role in localizing proteins at the nuclear periphery. Within the Apicomplexa, these two pathways seem restricted to a few species closely related to *Toxoplasma*.

In contrast, both *Toxoplasma* and *Plasmodium* possess conserved *C*-mannosylation and *O*-fucosylation pathways for modification of TSRs. These repeats are present in key surface adhesins and are possibly acquired by horizontal transfer from an animal source. As in animals, *C*-mannosylation and *O*-fucosylation of TSR occur in the early secretory pathway and seem to stabilize these repeats.

The presence of simple *O*- and *C*-glycans in Apicomplexans proteins has now been clearly established (Figs 4 and 5). Future work will likely clarify the nature of the proteins carrying these modifications and explore the importance of glycosylation for the parasite biology and pathogenicity.
